# Sexual Health

**Published:** 1891-11

**Authors:** 


SEXUAL HEALTH: A Companion to *Modern Domestic Medicine*. By Henry G. Hanchett, M. D., F. A. A., Member New York State and County Homœopathic Medical Societies; formerly Staff Physician to the College and Wilson Mission Dispensaries; Fellow of the New York Academy of Anthropology; Member Anglican Historical Association, etc., etc. Carefully revised by A. H. Laidlaw, A. M., M. D. Third edition. Philadelphia: The Hahnemann Publishing House, 1891.

This little manual of Dr. Hanchett has reached its third edition. Put into the hands of sensible parents it will surely do a vast amount of good. Boys and girls from sixteen to eighteen years of age may safely read it.

In speaking of the glans penis and a constricted foreskin, the author says: "We find the sources of many of the nervous disorders which are known to be caused by a long or tight foreskin, and among which are troubles of every sort in all parts of the body, including wetting the bed, *stammering*, twitchings, headache, epilepsy, and even something like hip disease; none of which troubles, when arising from a long foreskin, can be permanently cured without first circumcising the patient." We have had quite some experience regarding phimosis and stammering. Having observed several cases of stammering and stuttering, we invariably found phimosis, and would advise our physicians to look out for this condition when coming in contact with speech difficulties.

The paper, printing, and binding are good. It is a chaste little book.

Buy it, brother. W. S.

